# Direct determination of three-phase contact line properties on nearly molecular scale

**DOI:** 10.1038/srep26111

**Published:** 2016-05-17

**Authors:** P. M. Winkler, R. L. McGraw, P. S. Bauer, C. Rentenberger, P. E. Wagner

**Affiliations:** 1 University of Vienna, Faculty of Physics, 1090 Vienna, Austria; 2 Environmental and Climate Sciences Department, Brookhaven National Laboratory, Upton, NY 11973, USA

## Abstract

Wetting phenomena in multi-phase systems govern the shape of the contact line which separates the different phases. For liquids in contact with solid surfaces wetting is typically described in terms of contact angle. While in macroscopic systems the contact angle can be determined experimentally, on the molecular scale contact angles are hardly accessible. Here we report the first direct experimental determination of contact angles as well as contact line curvature on a scale of the order of 1nm. For water nucleating heterogeneously on Ag nanoparticles we find contact angles around 15 degrees compared to 90 degrees for the corresponding macroscopically measured equilibrium angle. The obtained microscopic contact angles can be attributed to negative line tension in the order of −10^−10^ J/m that becomes increasingly dominant with increasing curvature of the contact line. These results enable a consistent theoretical description of heterogeneous nucleation and provide firm insight to the wetting of nanosized objects.

The interaction of a solid substrate with a (partially) wetting liquid and a gas plays a crucial role in natural processes and technical applications. In biological systems, poor wetting properties are essential for any flying animal such as insects as they depend on proper wing run-off^1^. In contrast (nearly) perfect wetting inside plants regulates capillary action and thus nutrient supply^2^. Recently, industrial coatings have been introduced to achieve superhydrophobic surfaces in order to suppress chemical reactions and improve self-cleaning^3^,^4^. Similarly, aerosol and cloud formation depend largely on the interaction between a condensing liquid and the nucleating site^5^. Young^6^ has found that this interaction can be characterized by a well-defined (macroscopic) contact angle between solid and liquid surfaces in the vicinity of the three-phase contact line. Contact angles in the macroscopic scale have been measured for various compounds using the telescope goniometer method^7^,^8^, the Wilhelmy balance method^9^,^10^, the capillary tube method^4^ and a few other techniques. In the intermediate micron size range contact angles have been measured using atomic force microscopy (e.g.^11^) and SEM imaging (e.g.^12^).

However, in sub-micron size systems Gibbs^13^ already pointed out that an additional force term due to the increased curvature of the contact line may distort the macroscopic contact angle. The nanoscale force balance accounting for line tension yields the microscopic contact angle Θ expressed by the generalized Young equation^14^,^15^


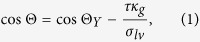


where Θ_*Y*_ is the macroscopic Young angle, *σ*_*lv*_ surface tension [J/m^2^], and *τ* line tension [J/m]. Here, *κ*_*g*_ refers to geodesic curvature of the contact line which is the curvature of this line viewed as a curve on the substrate surface. Detailed information on the nanoscale force balance and geodesic curvature together with a derivation based on Riemann geometry is provided in the online supplementary information. For the case of spherical substrate surface and circular contact line *κ*_*g*_ can be written in the form


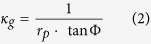


where *r*_*p*_ is the radius of a spherical substrate. Angle Φ manifests geodesic curvature and is illustrated in Fig. 1. Knowledge of Φ allows immediate determination of the geodesic curvature of the contact line. Consequently, for spherical geometry Eq. (1) can be expressed as^16^


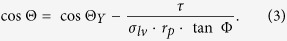


For a decreasing line tension term the microscopic contact angle approaches the macroscopic one. Only for sufficiently strong contact line curvature will wetting be influenced by the action of line tension. Modeling efforts indicate that this quantity is expected to be extremely small in the order of 10^−11^ J/m [15 and references therein]. It is therefore not surprising, that controversial experimental values of *τ* ranging from 3.10^−12^ J/m to >1.10^−5^ J/m are found in the literature^17^,^18^. Here we show that direct determination of microscopic contact angle Θ and angle Φ allows quantitative determination of *τ* if macroscopic contact angle and surface tension are known for a specific system.

Formation of aerosols and clouds is related to vapor-to-liquid nucleation processes^19^. In heterogeneous nucleation critical molecular clusters appear on particle surfaces. A schematic of a newly formed cluster on a seed particle with radius *r*_*p*_ can be seen in Fig. 1. Applying a macroscopic liquid drop concept, Fletcher^20^ has used a contact angle to approximately describe the interaction between clusters and the underlying particle surface. The Fletcher theory has been applied to a number of different compounds. Experiments on heterogeneous nucleation of water vapor on Ag nanoparticles resulted in large discrepancies from the Fletcher theory, if the macroscopically measured Young angle is used in the calculations^21^. Notably, these deviations can be strongly reduced by choosing a smaller value for the contact angle^22^. This observation indicates that a microscopic contact angle relevant for heterogeneous nucleation can be significantly different from the macroscopic Young angle.

To obtain quantitative experimental information on microscopic contact angles more detailed knowledge about the critical clusters formed at the particle surface during heterogeneous nucleation is required. The (excess) number n^*^ of molecules in a critical cluster can be directly determined applying the first nucleation theorem^23^,^24^


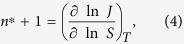


where *J* represents the nucleation rate measured as function of the saturation ratio *S* at constant temperature *T*. Equation (4) is a direct consequence of the law of mass action and quite generally valid independent from the assumptions of the classical nucleation theory. While homogeneous nucleation rates *J* are experimentally accessible [e.g.^25^], in heterogeneous nucleation experiments the quantity of prime interest is the nucleation probability *P* telling the fraction of preexisting particles that are activated by heterogeneous nucleation [e.g.^26^]. Correspondingly Eq. (4) has been reformulated for heterogeneous nucleation to obtain the (excess) number n^*^ of molecules in a critical embryo formed on the particle surface^27^


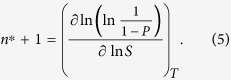


Under the assumption of incompressibility of the condensing liquid the volume of a critical cluster can be directly determined as v^*^ = n^*^ *v*_*l*_ , where n^*^ is obtained from Eqs (4) or (5) and *v*_*l*_ is the molecular volume of the bulk liquid.

Furthermore, the radius of curvature r^*^ of a critical cluster is given by the Kelvin relation^28^


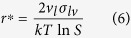


describing equilibrium vapor pressure conditions over curved surfaces. Here *S* represents the saturation ratio of the condensing vapor, *T* is the system temperature and *k* Boltzmann’s constant. The Kelvin relation has been evaluated by experiments on homogeneous [e.g.^25^] as well as heterogeneous^29^ nucleation and its predictions have been found to agree within experimental error for various compounds and cluster radii down to a scale of 1 nm. This result is supported by recent molecular dynamics simulations on critical cluster formation for Lennard-Jones molecules^30^,^31^, which result in surprisingly good agreement with the Kelvin relation.

Based on the above critical cluster properties the (microscopic) contact angle Θ, angle Φ, and hence geodesic curvature *κ*_*g*_ of the contact line can be directly determined. We consider heterogeneous nucleation of a supersaturated vapor on nearly spherical particles with radius *r*_*p*_ at the onset saturation ratio *S*_*onset*_, at which 50% of the particles are activated during a certain nucleation time period. From the nucleation theorem (5) we can determine the number n^*^ of molecules in the critical embryo and hence the critical embryo volume v^*^. From the Kelvin relation (6) we obtain the radius of curvature r^*^ of the critical embryo corresponding to the saturation ratio *S*_*onset*_. As can be seen from the cluster geometry shown in Fig. 1, knowledge of *r*_*p*_, r^*^ and v^*^ = n^*^ *v*_*l*_ is sufficient for a unique determination of the contact angle Θ and angle Φ (see Methods section).

We have determined microscopic contact angles Θ and angles Φ from experimental data on heterogeneous nucleation of water vapor on Ag particles^32^. The experimental procedure has been described in detail elsewhere [e.g.^22^]. In brief, Ag particles are produced in a tube furnace from the evaporation of silver wool at ~1300 K and subsequent homogeneous nucleation due to abrupt cooling. The polydisperse primary particle size distribution is then classified by a differential mobility analyzer (DMA) to obtain a monodisperse aerosol fraction at known size. Particles are then mixed with water vapor and led into the size analyzing nuclei counter (SANC) where a well-defined and uniform supersaturated vapor is achieved by adiabatic expansion. By scanning the vapor supersaturation from zero to full particle activation the number of activated particles is measured relative to the total particle number concentration, thereby yielding heterogeneous nucleation probabilities. In Fig. 2 heterogeneous nucleation probabilities *P* are shown as functions of vapor saturation ratio *S* for nucleation of water vapor on monodispersed Ag particles. Measurements were done at a nucleation temperature of 278 K using mobility diameters of 3.5, 5.5 and 7.0 nm. The corresponding *geometric* particle diameters relevant for the present study are smaller than the *mobility* diameters by 0.3 nm^33^. In order to verify the shape of the particles we collected particles right after the tube furnace on a silicon nitride grid and analyzed them in a transmission electron microscope (TEM). The electron micrograph in Fig. 3 shows the Ag particles used in this study and an approximately spherical shape of the particles can be observed.

Results of the evaluations are presented in Table 1 and shown to scale in Fig. 4. It can be seen that the microscopic contact angles obtained are considerably smaller than the corresponding macroscopically measured equilibrium Young angle Θ_*Y*_ of approximately 90° 22. Remarkably, we now have all quantities available to determine line tension. By inserting Θ_*Y*_, Θ and Φ into Eq. (3) line tension *τ* is found to be −(0.94 ± 0.1) · 10^−10^ J/m on average, accounting only for experimental uncertainties resulting from the heterogeneous nucleation experiments. However, in order to apply Eq. 3 properly we need to assume comparable surface properties, i.e., similar degree of oxidation between the silver plates used in the macroscopic contact angle measurements and the laboratory-generated nanoparticles studied here. We therefore allowed Θ_*Y*_ to vary between 70° and 110° to ensure that the appropriate macroscopic contact angle is considered in the evaluation. Apparently, this variation analysis translates into a larger uncertainty range for the line tension yielding *τ* = −(0.94 ± 0.3) · 10^−10^ J/m. This uncertainty range is thus primarily a consequence of the unknown “real” Θ_*Y*_ value and is not associated with our method for the determination of microscopic contact angle and contact line curvature. It should be emphasized here that we do not need to assume any degree of oxidation for the silver nanoparticles in order to obtain microscopic contact angles. The obtained microscopic contact angles provide an integral measure for the interaction of water with the corresponding surface.

Interestingly, we find negative values for line tension which also have been predicted by Gibbs^13^. In fact, a recent modelling study of Lennard-Jones nanodrops reports that negative values of line tension lead to reduced microscopic contact angles and that line tension is the dominant mechanism for the size dependence of the contact angle^34^. It is also worth noting that the ratio *σ*_*lv*_/*τ* (see Eqs 1 and 3) provides a characteristic measure of contact line curvature and is found to be on the order of ~1 nm^−1^. This is the scale where line tension effects on contact angle come into play. A negative line tension indicates strong seed-cap interaction at this scale that favors wetting of the seed and leads to smaller contact angles, as obtained in the present study from experimental nucleation data. A more detailed interpretation based on nucleation theory depends on the specific molecular properties of the considered compounds and will be the subject of a future publication.

While we find negative values of line tension for the system water-silver, line tension may also have positive values for different systems and is sensitive to temperature^35^,^36^. It is worth noting though that the absolute value of *τ* found in our study agrees nicely with recent experimental studies based on atomic force microscopy^37^ and fits well to theoretical predictions^38^.

Our study clearly shows that three-phase contact line properties such as contact line curvature, microscopic contact angle and line tension can be directly determined on a scale of 1 nm. The evaluation based on heterogeneous nucleation experiments is not reliant on nucleation theory and only requires incompressibility of the condensing liquid. Besides several other applications microscopic contact angles are important for consistent use in heterogeneous nucleation theory. So far the contact angle has often been considered as a free parameter in heterogeneous nucleation theory. In some experiments the macroscopically measured Young angle was used^21^, in other studies the contact angle was varied in order to fit Fletcher theory to experimental data^22^. Generally, these approaches result in inconsistencies or even large discrepancies from corresponding experimental data^21^. Use of the microscopic contact angle and the line tension as derived in this study enables an improved geometrically consistent theoretical description of heterogeneous nucleation on (partially) wettable particles.

It should be noted that contact angles and line tensions derived above were obtained for spherical geometry. On the molecular scale deviations from an ideal spherical shape are likely to occur. In this case the effective contact angle and line tension obtained can be viewed as a surrogate for surface interaction. The fact that the microscopic contact angles differ from the Young (macroscopic) contact angle is likely due to microscopic interactions that occur on the molecular scale and can be attributed to line tension. Line tension should therefore be carefully considered during the initial steps of phase transition on nanosized objects such as in nanoparticle and cloud formation.

## Methods.

For determination of the contact angle Θ we consider the ratio v^*^/v_dr_^*^ of the volume v^*^ = n^*^ *v*_*l*_ of the critical spherical cap embryo and the volume *v*_dr_^*^ = (4π/3)r^*^^3^ of a spherical drop with radius r^*^. This volume ratio can be expressed in terms of *x* = *r*_*p*_/r^*^ and *m* = cos Θ by the geometric equation^20^,^27^


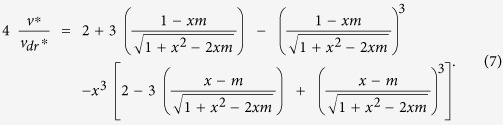


The term *m* can be determined as the real root of this equation and from *m* the contact angle Θ is obtained.

In the same notation angle Φ is given by^20^


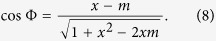


Heterogeneous nucleation probabilities are considered around the onset saturation ratio *S*_*onset*_. We are expressing the nucleation probability *P*(*S*) around *S*_*onset*_ in the form of the two-parameter fit function


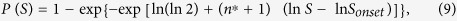


which has the form of a cumulative Gumbel distribution^39^. As shown in the online supplementary information this function comes naturally from the first nucleation theorem. Notably the fit parameters n^*^ and *S*_*onset*_ are related to the slope of *P*(*S*)


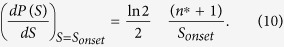


**Figure 2:**
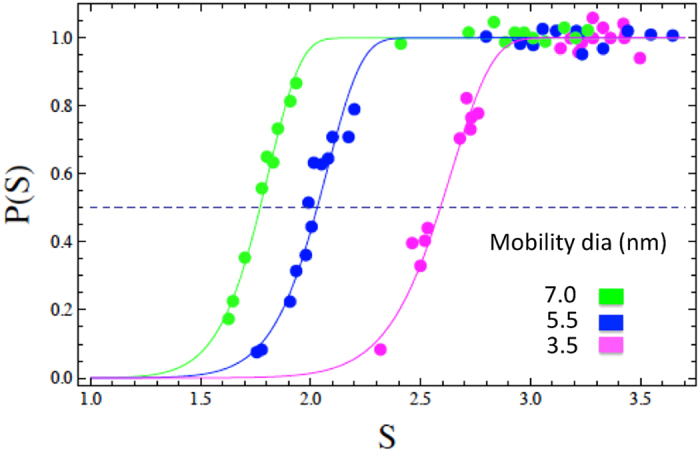
Heterogeneous nucleation probabilities^32^ and corresponding model fits (Eq. 9). The dashed line indicates 50% of activated particles from which corresponding onset saturation ratios *S*_*onset*_ can be obtained. The fit parameters n^*^ and *S*_*onset*_ are given in Table 1.

A least squares fit of the above function *P(S)* to the experimental nucleation probabilities simultaneously provides *S*_*onset*_ and n^*^. The corresponding fit curves are shown in Fig. 2. From *S*_*onset*_the critical radius r^*^ is calculated according to the Kelvin relation, Eq. (6), from n^*^ the critical embryo volume v^*^ = n^*^ *v*_*l*_ is obtained. The microscopic contact angle Θ can now be determined by inserting *r*_*p*_, r^*^ and v^*^ into Eq. (7) and solving for *m* = cos Θ. Angle Φ can be obtained by means of Eq. (8). The effect of the finite width of the particle size distribution (σ_g_ = 1.05) has been accounted for [40 and supplementary information].

## Additional Information

**How to cite this article**: Winkler, P. M. *et al*. Direct determination of three-phase contact line properties on nearly molecular scale. *Sci. Rep.* **6**, 26111; doi: 10.1038/srep26111 (2016).

## Supplementary Material

Supplementary Information

## Figures and Tables

**Figure 1:**
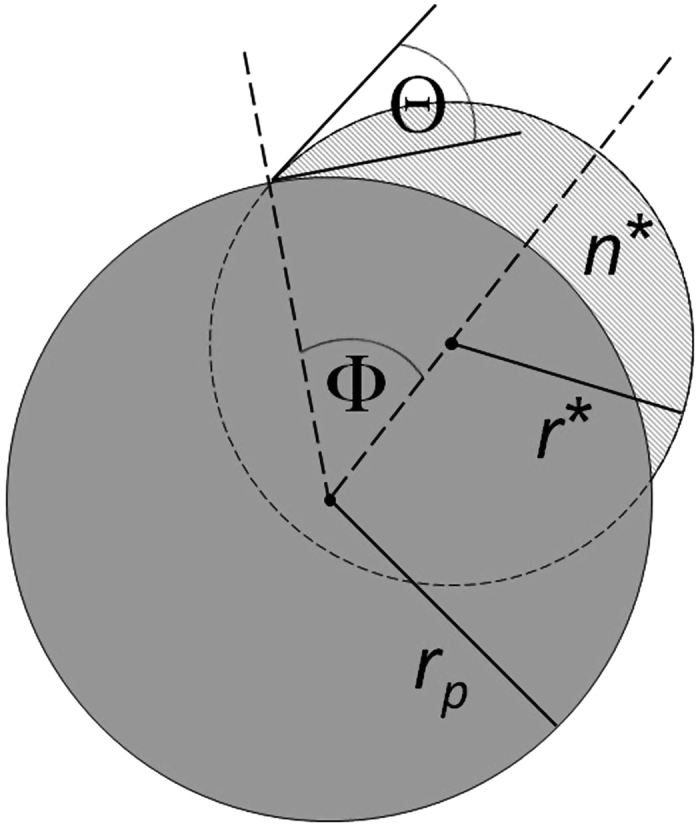
2D-schematic illustrating the geometry of a cluster when formed on a seed particle with radius *r*_*p*_. The newly formed cluster contains n^*^molecules and is characterized by the corresponding radius of curvature r^*^ from the Kelvin relation (see main text for detailed explanations). Interaction between cluster and particle surface leads to corresponding contact angle Θ and angle Φ.

**Figure 3:**
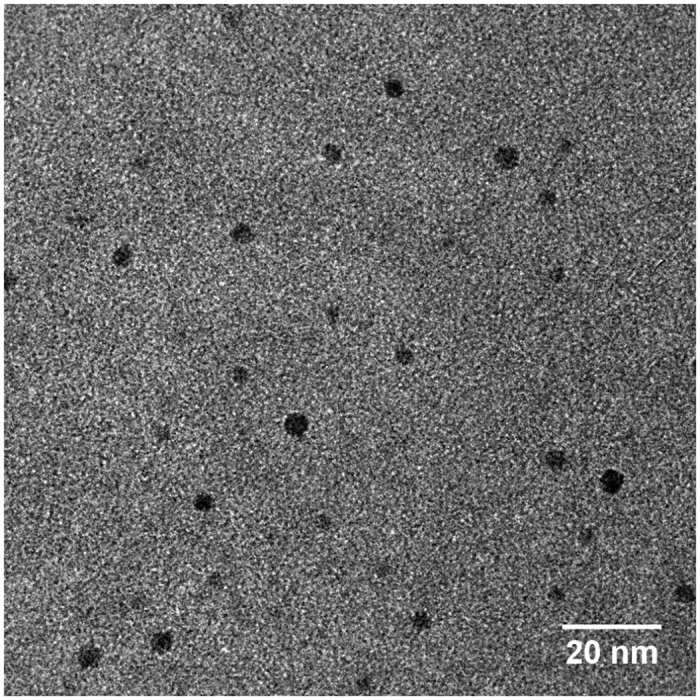
Electron micrograph of Ag particles from the tube furnace prior to size selection enlarged by a factor of 125 k. At the particle generator operating conditions used the primary particle size distribution exhibits a maximum at 4.8 nm and covers the entire range of particle sizes considered in this study. The particles found in the TEM picture agree nicely with the expected size range and clearly show spherical shape.

**Figure 4:**
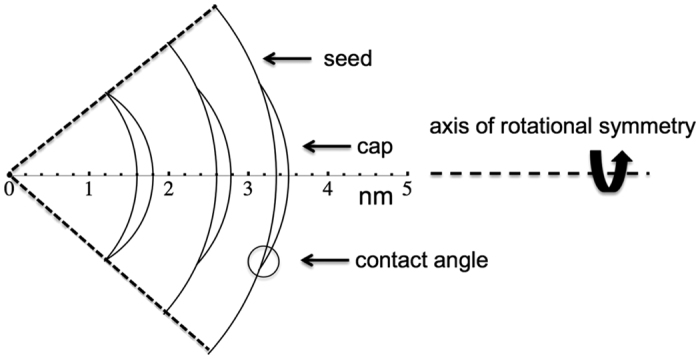
Cluster geometry shown to scale. Distances in nm. Left to right, particle (Ag) geometric diameters of 3.2, 5.2, and 6.7 nm with corresponding cap (H_2_O) radii of curvature and contact angles corresponding to the entries in Table 1.

**Table 1:** Results of evaluations for water clusters on Ag nanoparticles at nucleation temperature 278 K. Calculations have been made using *σ*_*lv*_ (278 K) = 74.96.10^−3^ J/m^2^ 41.

***r***_***p***_ **(nm)**	**S**_**onset**_	r^*^ **(nm)**	n^*^	**Θ**	**Φ**	***τ*** **(J/m) (Θ**_***Y***_** = 90°)**
1.6	2.59 ± 0.006	1.23	14.7 ± 1.5	18.0 ± 0.7	41.5	−1.01·10^−10^
2.6	2.03 ± 0.011	1.65	13.5 ± 1.1	17.3 ± 0.5	25.6	−0.89·10^−10^
3.35	1.77 ± 0.014	2.05	12.8 ± 0.7	14.7 ± 0.3	20.8	−0.92·10^−10^
