# Supplementary material for: Direct determination of three-phase contact line properties on nearly molecular scale
Source: Sci Rep. 2016 May 17;6:26111. doi: 10.1038/srep26111 (PMC4869068; doi:10.1038/srep26111)
Supplement: Supplementary Information [file srep26111-s1.pdf]

SUPPLEMENTARY INFORMATION for the manuscript entitled

Direct determination of three-phase contact line properties on nearly molecular scale

by P.M. Winkler, R.L. McGraw, P.S. Bauer, C. Rentenberger, P.E. Wagner

S1: Procedure for fitting nucleation probability measurements

From measurements of heterogeneous nucleation probability *P(S)* as a function of vapor saturation ratio, *S*, we seek to determine both the onset saturation ratio, S_{onset}, defined such that $P(S_{onset})$ =1/2, and n^*, which is related to the slope $(dP/dS)_{S=S_{onset}}$. *P(S)*, Eq. 9, can also be written as [McGraw et al., 2012, Eqs. 12-14]:

$$P(S)=1-\frac{N(t)}{N(0)}=1-e^{-J_1(S)t} \qquad \text{S1.1}$$

where *N(t)* is the concentration of unactivated particles at a residence time *t* defined by the operating conditions of the SANC. $J_1(S)$, units s^{-1}, is the heterogeneous nucleation rate per unactivated seed particle. From these considerations it follows that $J_1(S_{onset})t=\ln(2)$. This result will be used to eliminate residence time from the equations below.

<u>Method 1: Nonlinear fit to *P(S)*</u>

A fitting procedure for *P(S)* derives naturally from the first nucleation theorem applied to the per seed nucleation rate:

$$\left(\frac{\partial \ln J_1}{\partial \ln S}\right)_T = n^* + 1 \qquad \text{S1.2}$$

This result requires no specific model of the nucleation process, only the fundamental principles of mass action and detailed balance, on which the nucleation theorem itself is based. Although the nucleation theorem is a differential result, much of its utility derives from local linearity of $\ln J_1$ as a function of $\ln S$ supported both by experiment and theory [Wolk and Strey, 2001; McGraw and Wu, 2003]. Figure S1 below supports this local linearity for the present heterogeneous nucleation measurements. Integrating at constant temperature:

$$J_1(S)t = J_1(S)\ln 2 / J_1(S_{onset})$$
$$\ln[J_1(S)t] = \ln(\ln 2) + (n^*+1)\{\ln(S) - \ln(S_{onset})\}$$

where the SANC residence time has cancelled from the final result. Exponentiating the last equation and substituting the result into Eq. S1.1 gives our final form for the nucleation probability distribution:

$$P(S) = 1 - \exp\left\{ -\exp\left[\ln(\ln 2) + \left(n^* + 1 \right) \left(\ln S - \ln S_{onset} \right) \right] \right\}. \qquad \text{S1.3}$$

The sought parameters S_{onset} and n^* are obtained from a nonlinear model fit and are related to the slope of *P(S)* at onset.

$$\left(\frac{dP}{dS} \right)_{S=S_{onset}} = \frac{(n^* + 1)\ln 2}{2 S_{onset}} \qquad \text{S1.4}$$

Method 2: Linear fit to ln(J_1 *t*) versus ln(*S*)

The linear form derived above from local extension of the nucleation theorem serves as a basis for linear regression in the variables ln (J_1 *t*) and ln *S*:

$$\ln[J_1(S)t] = a + b\ln(S) \qquad \text{S1.5}$$

Comparison with Eq. S1.3 shows $n^* + 1 = b$ and $\ln S_{onset} = \left[\ln(\ln 2) - a\right] / b$. The original data set $\{S_i, P(S_i)\}$ is first transformed to $\{\ln S_i, \ln[J_1(S_i)t]\}$ with the aid of Eq. S1.1. Conventional linear least squares analysis is next used to obtain *a* and *b*, from which n^* and $\ln S_{onset}$ are determined by the relations given above. This approach has the advantage that it is easier to perform subsequent error analysis but requires a screening criterion to eliminate *P(S)* values either close to zero or in the saturation limit of Fig. 2, which need to be removed for a proper fit. The resulting fit is illustrated in Fig. S1 for three size classes at *T* = 278*K*.

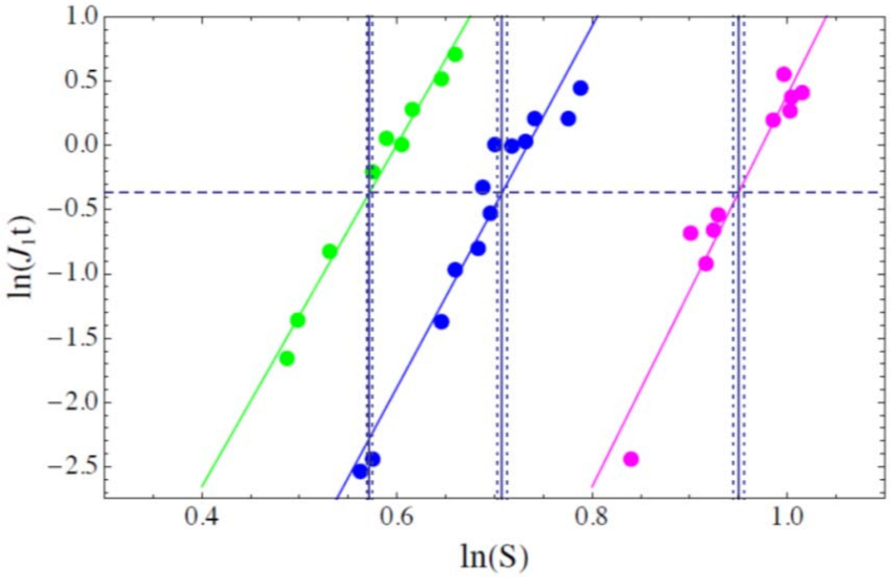

Fig. S1. Linear fits to the exponent of Eq. S1.1. Results at the three particle sizes and T = 278K. Vertical lines show the mean and mean ± σ values of ln S obtained as described in the text.

Measurements having either *P(S)* >0.9 or zero were removed form data, so as to avoid saturation and infinitely negative values of the ordinate. The measurements included in the figure span about one order of magnitude in nucleation rate. The new estimates for n^* and $\ln S_{onset}$, are those given in Table 1. These differ negligibly from the values (not reported here) obtained from the nonlinear model fit.

S2. Analysis of uncertainty

Because the saturation ratios are determined accurately in the SANC (Winkler et al., 2008), it is assumed here that the largest uncertainty lies in the measurements of $\ln[J_1(S_i)t]$. Because the measurements employ the same instrument and expansion ratio, which is held constant at constant *T*, it is further assumed that the variance in each measurement is the same throughout a given size class data set. Uncertainties in the slope and intercept parameters of Eq. S1.5 and in the derived physical parameters:

$$n^* = b - 1 \text{ and } \ln S_{onset} = \{\ln[\ln(2)] - a\} / b \qquad \text{S2.1}$$

are obtained following the procedures given in *Numerical Recipes* [Press et al., 1992]. Determining uncertainty in $\ln(S_{onset})$, and therefore in S_{onset}, follows standard propagation of error:

$$\sigma^2_{\ln S_{onset}} = \sigma_a^2 \left(\frac{\partial \ln S_{onset}}{\partial a}\right)^2 + \sigma_b^2 \left(\frac{\partial \ln S_{onset}}{\partial b}\right)^2 + 2\sigma_{ab}^2 \left(\frac{\partial \ln S_{onset}}{\partial a}\right)\left(\frac{\partial \ln S_{onset}}{\partial b}\right) \qquad \text{S2.2}$$

where σ^2 is error variance. Note that because errors in *a* and *b* are strongly anti-correlated it is important to include the covariance term on the right in Eq. S2.2. The uncertainty ranges obtained from this analysis are reported in Table 1, and shown graphically by the spacings between the dotted vertical lines of Figure S1.

S3. Effects from seed particle polydispersity

The finite width of the seed particle size distribution has been estimated as having $\sigma_g = 1.05$ where σ_g is the geometric standard deviation. Accordingly, each seed particle, of (spherical) diameter *d*, within a size class can be expected to have a slightly different nucleation probability curve. Allowing for size dependence in Eq. S1.3 gives:

$$P\left(S, d, n^*\right) = 1 - \exp\left\{ -\exp\left[\ln(\ln 2) + \left(n^* + 1 \right) \left(\ln S - \ln S_{onset}(d) \right) \right] \right\} \qquad \text{S3.1}$$

The function $S_{onset}(d)$ is determined by fitting the onset saturation ratios for the three main particle size classes given in Table 1. Any size dependence in n^* lies within the uncertainty range of this quantity and is neglected; this is supported, qualitatively, through inspection of Fig. S1, which shows significant shift in onset saturation ratio with seed size class but little change in slope.

Integrating Eq. S3.1 over the normalized seed particle size distribution yields the nucleation probability curve for the polydisperse case:

$$P_{poly}(S) = \int_0^\infty P(S, x, \kappa_1) p(x) dx \qquad \text{S3.2}$$

where for clarity of notation the seed particle diameter is now *x*. *p(x)* is the seed distribution, assumed here to be lognormal. Calculations were carried out for the intermediate seed-size class (5.2 nm). The geometric mean diameter to also set at this value. The geometric standard deviation is assigned the value σ_g=1.05. Comparison of S1.3 and S3.2 (not shown) shows the leading effect of polydispersity to be a broadening of the nucleation probabilty curve, with systematic reduction in its slope, relative to the monodisperse case, but little change in S_{onset}. The smaller slope implies that polydispersity results in a slight but systematic underestimatation of critical nucleus size, because restoring agreement with the measurements requires increasing n^* in Eq. S3.2 to sharpen the region of nucleation onset back to the steepness of the original (monodisperse) fit.

This correction turns out to be remarkably small: For $d = 5.2nm$, and σ_g =1.05, it was found that increasing the slope parameter from n^* = 13.0 in Eq. S3.1 to n^* = 13.5 in Eq. S3.2 restored the fit. To put this another way, the fits obtained from Eq. S1.3 with n^* = 13.0 and from Eq. S3.2 with n^* = 13.5 were visually identical. Both

fits are in agreement with the measurements but because polydispersity is known to be present, the fit based on Eq. S3.2 has the greater physical justification. The result is that the *n** values in Table 1 were increased by 0.5 over the monodisperse values in order to take polydispersity into account.

The polydispersity correction will not always be small, especially if the nucleation onset probability distribution is steplike. Predicted activation characteristics of neutral particles were described by del la Mora (2011) for different ranges of the nucleation barrier height and n*. In the step limit (high barrier height, large n^*) the right hand side of Eq. S3.2 reduces to the cumulative distribution of *p(x)* - transformed to the *S*-coordinate. Polydispersity thus sets a lower limit on the width of the nucleation onset probability, which is realized in the limit of large n* and high, steplike, slope. For this case, even small seed polydispersity will result in significant underestimates of n^* relative to its large monodisperse value.

S4. Generalized Young equation and geodesic curvature

In a system consisting of a solid substrate, a partially wetting liquid and a gas we consider a contact point in the three-phase contact line. The wetting behaviour and particularly the contact line at the considered three-phase contact point are depending on a force balance in the plane tangential to the substrate surface at this contact point.

For a straight contact line on a plane substrate surface we consider the forces corresponding to the interfacial tensions σ_{sv} , σ_{sl} , σ_{lv} , where *s, l* and *v* denote solid, liquid and vapour, respectively. From force balance in the plane substrate surface Young [Young, 1805] obtained the equation

$$\cos\Theta_Y = \frac{\sigma_{sv} - \sigma_{sl}}{\sigma_{lv}} \qquad \text{(S4.1)}$$

for the (macroscopic) Young angle Θ_Y .

Gibbs [Gibbs, 1878] already pointed out that for the case of a curved contact line the influence of an additional force corresponding to a line tension needs to be considered. In the general case of a curved substrate surface this line tension force in the considered tangent plane can be expressed in the form [Boruvka&Neumann (1977), Pompe&Herminghaus (2000)]

$$f_l = \tau \kappa_g , \qquad \text{(S4.2)}$$

where the geodesic curvature κ_g is the curvature of the contact line *seen in the curved substrate surface* and τ is the line tension. Actually κ_g is a measure for the deviation of the contact line from a geodesic curve in the substrate surface. If the contact line is a geodesic in the substrate surface, the corresponding geodesic curvature is zero and no line tension force in the tangent plane occurs. If the line tension force f_l is included into the above mentioned force balance in the tangent plane the generalized Young equation [Boruvka&Neumann (1977), Pompe&Herminghaus (2000)]

$$\cos\Theta = \cos\Theta_Y - \frac{\tau}{\sigma_{lv}}\kappa_g \qquad \text{(S4.3)}$$

is obtained, where Θ denotes the (microscopic) contact angle and the (macroscopic) Young angle Θ_Y is expressed by Eq. (S4.1). As indicated by Gibbs [Gibbs, 1878] the line tension τ can be negative.

In the following we consider the case of a spherical solid substrate surface with radius r_p and a circular contact line with radius (see Fig. S2)

$$\rho = r_p \sin\Phi\,. \qquad \text{(S4.4)}$$

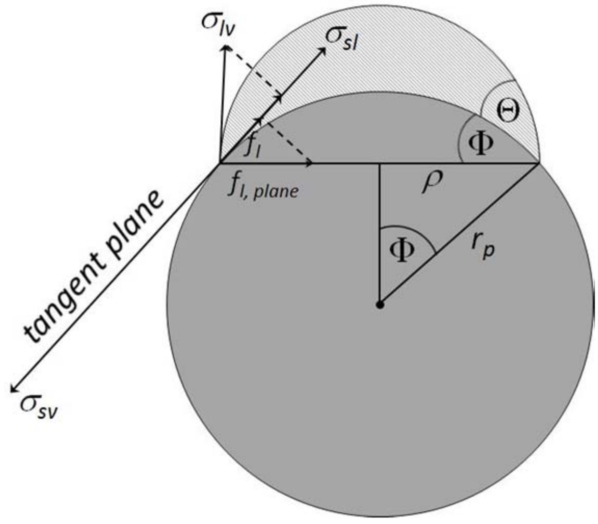

Fig. S2. Spherical wetting geometry

The force balance

$$\sigma_{sv} = \sigma_{sl} + \sigma_{lv} \cos\Theta + \tau\,\kappa_g \tag{S4.5}$$

in the tangent plane is illustrated in Fig. S2. Here $\tau\,\kappa_g = f_l$ is the line tension force in the tangent plane. While the (ordinary) curvature κ of the contact line seen in the plane of the contact line would refer to a line tension force $f_{l,\,plane} = \tau\,\kappa$ in the plane of the contact line, the geodesic curvature κ_g corresponds to the orthogonal projection f_l of this line tension force onto the tangent plane (see Fig. S2) and accordingly

$$\kappa_g = \kappa \cos\Phi\,. \tag{S4.6}$$

The (ordinary) curvature of the contact line seen in the plane of the contact line can be expressed as $\kappa = 1/\rho$ (see Eq. (S4.8) below). Accordingly Eq. (S4.6) yields

$$\kappa_g = \frac{1}{\rho}\cos\Phi = \frac{1}{r_p\,\tan\Phi}\,. \tag{S4.7}$$

In the following the curvatures κ and κ_g of the contact line are calculated applying Riemann geometry. The curvature of a curve can be obtained as the norm of the main normal vector of the curve. In the considered Riemann space with coordinates q^i, metric tensor $g_{i\,j}$ and Christoffel symbols $\Gamma^{\,i}_{m\,n}$ the curve has the parameter representation

$$q^i = q^i(s),$$

where the arc length *s* is the parameter and we can calculate the *unit* tangent vector

$$T^i = \frac{d\,q^i}{d\,s}\,.$$

Now the absolute (covariant) derivative of $\vec{T}$ along the curve yields the main normal vector

$$N^i = \frac{DT^i}{Ds} = T^i{}_{|j} \frac{dq^j}{ds} = \frac{d^2q^i}{ds^2} + \Gamma^i_{m\,n} \frac{dq^m}{ds} \frac{dq^n}{ds} .$$

Finally the curvature of the curve is obtained as

$$\kappa = \left| \vec{N} \right| = \sqrt{g_{i\,j}\, N^i\, N^j} \;.$$

For calculation of the (ordinary) curvature κ of the circular contact line *seen in the plane of the contact line* we consider Cartesian coordinates $(x^i) = (x, y)$ in the plane of the contact line. For Cartesian coordinates we have $g_{i\,j} = \delta_{i\,j}$ and $\Gamma^i_{m\,n} = 0$. The contact line with radius $\rho = r_p \sin \Phi$ has the parameter representation

$$\begin{cases} x = \rho \cos \dfrac{s}{\rho} \\ y = \rho \sin \dfrac{s}{\rho} \end{cases}$$

where the parameter *s* is the arc length of the contact line. The unit tangent vector

$$\left(T^i \right) = \begin{pmatrix} \dfrac{dx}{ds} \\ \dfrac{dy}{ds} \end{pmatrix} = \begin{pmatrix} -\ \sin \dfrac{s}{\rho} \\ \cos \dfrac{s}{\rho} \end{pmatrix}$$

is calculated and further we get the main normal vector

$$\left(N^i \right) = \begin{pmatrix} \dfrac{d^2x}{ds^2} \\ \dfrac{d^2y}{ds^2} \end{pmatrix} = \begin{pmatrix} -\dfrac{1}{\rho} \cos \dfrac{s}{\rho} \\ -\dfrac{1}{\rho} \sin \dfrac{s}{\rho} \end{pmatrix}.$$

Now we obtain the (ordinary) curvature κ of the circular contact line *seen in the plane of the contact line* as the norm of $\vec{N}$:

$$\kappa = \left| \vec{N} \right| = \frac{1}{\rho} = \frac{1}{r_p \sin\Phi}. \tag{S4.8}$$

For calculation of the curvature of the circular contact line *seen in the spherical substrate surface,* which is the geodesic curvature κ_g, we consider spherical coordinates $(q^i) = (\varphi, \alpha)$ in the spherical substrate surface. For these coordinates we have the metric tensor

$$\left(g_{ij}\right) = \begin{pmatrix} r_p{}^2 & 0 \\ 0 & r_p{}^2 \sin^2\varphi \end{pmatrix}$$

and the Christoffel symbols

$$\left(\Gamma^1_{i\,j}\right) = \begin{pmatrix} 0 & 0 \\ 0 & -\sin\varphi\cos\varphi \end{pmatrix}, \quad \left(\Gamma^2_{i\,j}\right) = \begin{pmatrix} 0 & \cot\varphi \\ \cot\varphi & 0 \end{pmatrix}.$$

The contact line with radius $\rho = r_p \sin\Phi$ has the parameter representation

$$\begin{cases} \varphi = \Phi \\ \alpha = \dfrac{s}{r_p \sin\Phi} \end{cases}$$

where the parameter s is the arc length of the contact line. The unit tangent vector

$$\left(T^i\right) = \begin{pmatrix} \dfrac{d\varphi}{ds} \\ \dfrac{d\alpha}{ds} \end{pmatrix} = \begin{pmatrix} 0 \\ \dfrac{1}{r_p \sin\Phi} \end{pmatrix}$$

is calculated and further we get the main normal vector

$$\left(N^i\right) = \left(\frac{d^2 q^i}{ds^2} + \Gamma^i_{m\,n} \frac{dq^m}{ds}\frac{dq^n}{ds} \right) = \begin{pmatrix} \dfrac{-1}{r_p{}^2 \tan\Phi} \\ 0 \end{pmatrix}.$$

Now we obtain the curvature of the circular contact line *seen in the spherical substrate surface,* which is the geodesic curvature

$$\kappa_g = \left| \vec{N} \right| = \frac{1}{\left| r_p \tan\Phi \right|} \ . \qquad \text{(S4.9)}$$

Eqs. (S4.8) and (S4.9) confirm the intuitive Eqs. (S4.6) and (S4.7).

S5. References:

Fernandez de la Mora, J., Aerosol Sci. Technol., **45**, 543-554 (2011).

McGraw, R., J. Wang and C. Kuang, Aerosol Sci. & Technol. 46, 1053-1064 (2012).

McGraw, R. and D. T. Wu, J. Chem. Phys., **118**, 9337-9347 (2013).

Press, W. H., S. A. Teukolsky, W. T. Wetterling, B. P. Flannery, *Numerical Recipes in FORTRAN* (Cambridge University Press, Cambridge, 1992) pg. 655.

Winkler, P. M., Vrtala, A. and Wagner, P. E., Atmos. Res., **90**, 125–131, 2008).

Wölk, J. and R. Strey, J. Phys. Chem. **B105**, 11683 (2001).

Young, T. *Phil. Trans. R. Soc. London* **95**, 65 (1805).

Gibbs, J. W., Trans. Connecticut Acad. III, pp.108 (1878).

Boruvka, L., Neumann, A. W., *J. Chem. Phys.* **66**, 5464 (1977).

Pompe, T., Herminghaus, S., *Phys. Rev. Lett.* **85**, 1930 (2000).
